# Branch spiral beam harvester for uni-directional ultra-low frequency excitations

**DOI:** 10.1016/j.heliyon.2024.e34776

**Published:** 2024-07-25

**Authors:** Iresha Erangani Piyarathna, Mustafa Ucgul, Charles Lemckert, Zi Sheng Tang, Yee Yan Lim

**Affiliations:** a Faculty of Science and Engineering, Southern Cross University, East Lismore, NSW, 2480, Australia; b College of Science and Engineering, James Cook University, 1 James Cook Drive, QLD, 4811, Australia; c Sri Emas International School, Shah Alam, 40000, Selangor, Malaysia

**Keywords:** Spiral branch beam harvester, Human motion, Macro-fibre composite (MFC), Piezoelectric energy harvesting, Optimisation, Box-behnken

## Abstract

In recent years, there has been a growing interest in piezoelectric energy harvesting systems, particularly for their potential to recharge or replace batteries in energy-efficient electronic devices and wireless sensor networks. Nonetheless, the conventional linear piezoelectric energy harvesters (PEH) face limitations in ultra-low frequency vibrations (1–10 Hz) due to their narrow operating bandwidth and higher resonance frequencies. To address this, researchers explored compact shaped geometries, with spiral PEH being one such design to lower resonance frequencies by reducing structural stiffness. While trying to achieve this lower resonance frequency, spiral designs overlooked that they were spreading the stress across the structure. This was a significant drawback because it reduced the structure's ability to stress the piezoelectric transducer. The issue remains unaddressed, limiting the power generation of spiral beam harvesters. Furthermore, spiral structures also fail to broaden the operating bandwidth, posing additional constraints on their effectiveness. This study introduces a novel solution – the “branch spiral beam harvester,” combining the benefits of both spiral and branch beam designs. The integration of the branch beam concept into the spiral structure aimed to broaden the effective frequency range and establish a concentrated stress area for the placement of the piezoelectric transducer. Finite Element Analysis (FEA) was employed to assess operating bandwidth and stress distribution, while experimental studies evaluated voltage and power generation. Once the workability was confirmed, a statistical optimisation method was introduced to tailor the harvester for specific frequencies in the ultra-low frequency range. Results indicated that the branch spiral beam harvester exhibits a wider operating bandwidth with six natural frequencies in the ultra-low frequency range. It harnessed significantly higher output voltages and power compared to conventional linear PEH. This innovation presents a promising advancement in piezoelectric energy harvesting, offering improved performance without the need for proof masses or additional accessories.

## 1 Introduction

During the last few decades, wireless sensor networks (WNS) have gradually increased as they are deployed in several applications, including smart electronic devices [1]. In most cases, to operate these smart devices sufficiently, it is necessary to have the connected wireless sensors available constantly. The biggest challenge is supplying continuous power to avail them all the time [2]. The batteries as the primary power source for these systems are not very promising. They encounter various drawbacks, including limited life span, frequent replacements, hazardous replacements, capacity and high-cost maintenance. Therefore, a reliable power supply such as small-scale renewable energy harvester is beneficial and essential for such advanced systems. Vibration energy is a readily available and widespread energy source in the surrounding environment. Smart devices are frequently exposed to low-frequency vibration sources, including wind, structural vibrations, vehicular motion, ocean waves, and human motion [3]. They can be converted into electrical energy with a proper transduction mechanism to replace the batteries in WNS.

Out of many mechanisms, the piezoelectric transduction mechanism has drawn significant attention for converting vibration energy into electrical energy due to its simple integration, high power density, scalability and minimal damping [4]. The conventional linear piezoelectric energy harvester (PEH) is a flexible cantilever beam attached to one or two piezoelectric transducers. The nominal working principle is that when the system vibrates, the cantilever beam deflection develops a strain near the fixed end, eventually converted into electrical energy by the piezoelectric transducer. The structure of a conventional PEH physically equals a single mass-spring-damper system with a single resonant frequency, at which the harvester works most effectively [2]. When the excitation frequency deviates from this resonance frequency, the output power of the harvester decreases significantly, emphasising a major drawback of the conventional PEH. Besides, the higher modes of the conventional PEH are typically not employed in energy generation since they are far from the fundamental frequency [5]. Therefore, conventional PEH is inapplicable for many practical applications due to its narrow operating bandwidth, as the ambient vibrations are spread over a broader frequency spectrum. Mainly, harvesting energy from ultralow frequency vibrations (1–10 Hz) [6], such as human motion using conventional PEH, is less productive and highly challenging.

Since then, researchers have been exploring various methods to enhance conventional PEH systems, aiming to optimise performance through improvements in electrical design, material enhancements, and structural modifications, thereby improving power harvesting efficiency and expanding their operational bandwidth [[7], [8], [9], [10], [11], [12]]. The most direct approach involved adding a large, solid block of mass to decrease the resonance frequency of the system [13]. However, this step was unfavourable for many applications, such as human motion and wind energy harvesting, as it reduced power density while adding unnecessary weight to the system [14]. Alternatively, researchers applied different techniques such as different shape geometries [15,16], multimodal harvester concept [[17], [18], [19]], bistable or tristable configurations [20,21] either to achieve low frequency, broader operating bandwidth and/or power efficiency in the PEHs. As for shape geometries, simple to complex structures have been proposed to optimise the PEH performance. Among them, zigzag and spiral systems have been studied under various factorial changes since they deliver a compact design, reducing the stiffness of the harvester without increasing the harvester length [16]. Both systems can significantly reduce the magnitude of the first resonance frequency compared to the same volumetric conventional PEH. Sharpes, Abdelkefi [16], Sharpes, Abdelkefi [22] and Abdelmoula, Sharpes [23] extensively studied the zigzag PEHs and optimised their power generation based on their mode shape analysis. It was found that using zigzag-shaped harvesters is more beneficial than using non-linear energy harvesters [24]. Karami, Yardimoglu [25] introduced an Archimedean spiral beam to PEH, which is composed of different radius beam sections. The study disclosed that vibration modes were predominantly torsional and opposite signs of stress directing to subsequent charge cancellation in piezoelectric transducers. Santos, Hobeck [26] analysed an orthogonal spiral structure for the PEH substructure. The studies of spiral geometries, achieving much lower resonance frequency than zigzag structures [25]. Even though the proposed shaped geometries outperformed than conventional PEH with having lower first resonance frequency, there is still need an improvement mainly in terms of operating bandwidth of these harvesters. In addition, it is worth noting that while aiming for low resonance frequency, spiral designs have overlooked that reducing stiffness was achieved by distributing stress over the structure [16]. This is a major drawback as it lessens the beam's ability to stress the piezoelectric transducer which eventually resulted in low power generation. The issue has not been addressed to date and that limits the power generation of spiral beam harvesters.

This study introduces a novel uni-directional shaped geometry that merges the benefits of spiral and branch beam concepts. The motivation behind incorporating the branch beam concept into the spiral structure is to extend the operating bandwidth of the PEH. This expansion enables compatibility with a wider range of frequencies, including ultra-low frequency vibrations such as human motion. Additionally, these branches not only enhance power output by improving deformation and coupling efficiency but also boost sensitivity, thereby enabling effective detection of low-level vibrations [5,17]. Hence, one of the key objectives of this study is to achieve a few closer resonance peaks in the ultra-low frequency range (1–10 Hz). In addition, the proposed beam shape is designed to achieve a higher concentrated stress structure in which the majority of stress in the main beam is concentrated into a nominal area where the piezoelectric transducer will be placed. This will address the issue mentioned above identified in spiral beam harvesters. These improvements are aimed to achieve without using a proof mass or additional accessories (i.e., no magnet/impact stopper penalty) but purely with shaped geometry. The unique geometry of this PEH is designed to capitalises on the inherent flexibility of the spiral structure while integrating branch beams to enhance mechanical resonance. By eliminating proof masses and other external accessories, this design aims to propose a PEH that is more compact, lightweight, and easier to integrate into various applications, especially those where space and weight are critical considerations, such as wearable devices. Finite element analysis (FEA) and experimental studies were used to validate the design. FEA was undertaken to analyse the operating bandwidth and stress distribution, while the experimental study aimed to evaluate the proposed design's voltage and power generation ability. It is worth noting that this study is focused on proving the workability of the proposed design with an experimental study. Thus, theoretical analysis can be considered for future research to improve the device further. Once the workability of the device is proved, a statistical optimisation method is introduced to design the proposed uni-directional harvester to work in targeted frequencies in the ultra-low frequency range.

## 2 Materials and methods

### 2.1 Design of branched spiral beam harvester

**Fig. 1:**
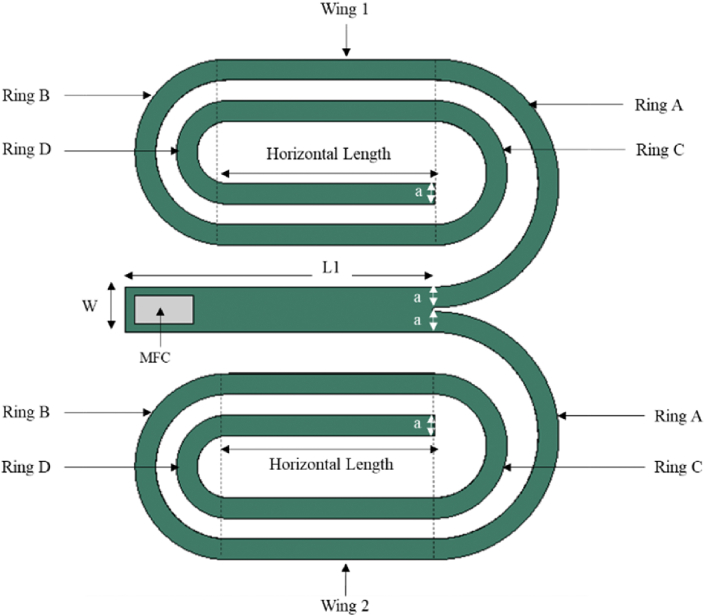
Schematic of proposed BSBH.

This study introduced a novel-shaped geometry named Branched Spiral Beam Harvester (BSBH) by combining a cantilever beam section with two spiral branch beam sections, as presented in Fig. 1. Herein, the proposed harvester is referred to as BSBH in the text. The cantilever beam performs as the main beam component of the design. A macro fibre composite (MFC - M2814 - P2 – (28 x 14 × 0.35 mm^3^)) piezoelectric transducer was attached near the fixed end of the main beam. The length of the main beam (L1 - 150 mm) was adopted from the previous spiral study presented by Tikani, Torfenezhad [27]. It is a known fact that the resonance frequency of the cantilever beam reduces with the increase in beam length [28]. However, an increase in beam length reduces the compactness of the structure. Introducing a spiral shape effectively extends the beam's length while maintaining a compact structure.

Consequently, the free end of the main beam in BSBH was designed with two identical spiral branch beams using the same aluminium plate. Anand and Kundu [29] found that turns in a spiral structure are a critical factor that influences the natural frequency of the harvester. Hence, to lower the natural frequency of the harvester, the maximum number of turns in the spiral was considered with respect to the main beam length while designing the BSBH. The geometrical properties of BSBH are labelled in Fig. 1 and summarised in Table 1. It is essential to highlight that the dimensions of the BSBH harvester were selected as a proof of concept. Given the unique properties of piezoelectric materials, this device can be scaled down to be used as microstructures for real-time applications. The physical properties of aluminium and MFC are summarised in Table 2, Table 3, respectively.

**Table 1:** Geometrical properties of BSBH.

Parameter	Dimension (mm)
L1	150
W	22
a	10
Inner Radius of Ring A	50
Inner Radius of Ring B	35
Inner Radius of Ring C	25
Inner Radius of Ring D	15
Outer Radius of Ring A	60
Outer Radius of Ring B	45
Outer Radius of Ring C	35
Outer Radius of Ring D	25
Horizontal Length	100

**Table 2:** Material properties of the Aluminium beam.

Parameters	Aluminium beam
Materials	Aerospace-graded Aluminium 2024
Elastic modulus (GPa)	70
Poisson's ratio	0.33
Density (kg/m^3^)	2780

**Table 3:** Material properties of MFC transducer.

Property	Young's modulus E1 (GPa)	Young's modulus E2 (GPa)	Poisson's ratio V12	Shear modulus G12 (GPa)	Piezoelectric charge constants d_33_ (pC/N)	Piezoelectric charge constants d_31_ (pC/N)	Active Area Density (kg/m³)
Value	30.336	15.857	0.31	5.515	4.6 × 10^2^	−2.1 × 10^2^	5440

**Fig. 2:**
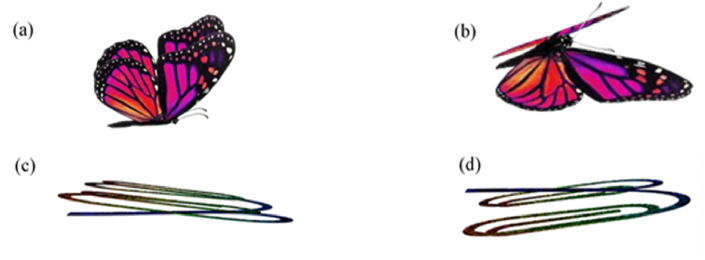
The dynamic motion of a butterfly and BSBH (a) Upstroke and (b) Downstroke of Butterfly (c) Upstroke and (d) Downstroke of BSBH.

The architecture of BSBH was inspired by the shape of a butterfly and its flapping of wings during forward flight. Butterflies create lift and thrust by upstroke (Fig. 2 (a)) and downstroke (Fig. 2 (b)) flapping of their wings [30]. At each stroke, the flapping frequency varies due to many factors [30,31] and ranges between 9 and 12 Hz [32]. The butterfly wings' size and shape help to create large wing deformations during lift and thrust [31]. These deformations generate immense aerodynamic forces abruptly in each upstroke and downstroke [33]. This phenomenon was used to design BSBH. However, it is worth noting that the air movement relative to the wing is irrelevant to this study. Conversely, the critical fact is while working under vibrations, the spiral branch beams of BSBH create large deformations and tend to amplify the deformation and bending of the main beam. Following this, the strain generated near the fixed end potentially increases, leading to generating high electrical output by the MFC transducers.

**Fig. 3:**
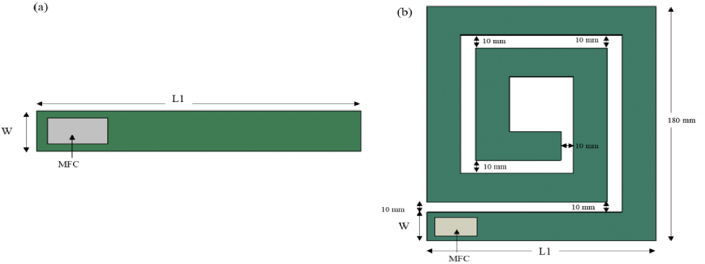
Schematics of PEH designs (a) CBH (b) SBH.

To evaluate the effectiveness of natural frequencies, operating bandwidth, stress distribution and mode shape analysis of BSBH, two existing beam designs, a conventional cantilever beam harvester (CBH, L1 – 150 mm, W – 22 mm) Figs. 3– (a)) and spiral beam harvester (SBH, L1 – 150 mm, W – 22 mm - Figs. 3– (b)), were also studied in FEA. To ensure fairness in our comparison, we maintained consistency by keeping the volume, main beam length, and the number of turns in the SBH the same as those in the BSBH. However, for the CBH equivalent volume concept was not considered as the length of the CBH becomes excessively long in this circumstance. Hence, the length of the CBH kept similar to the main beam length of both BSBH and SBH. The material properties of both designs were identical to BSBH and summarised in Table 2, Table 3

### 2.2 Finite Element Analysis (FEA)

To investigate the structural and mechanical characteristics of the proposed BSBH, Finite Element Analysis (FEA) was conducted utilising the commercially available software package SIMULIA ABAQUS™. The resultant simulations facilitated the comprehensive assessment of critical parameters, encompassing stress distribution, stress-strain generation, proximity of natural frequencies, operating bandwidth, and mode shapes of the BSBH, against the evaluations of the CBH and SBH. This rigorous analysis of stress and strain generation within the harvester systems served as a predictive measure of their potentiality for voltage and power generation.

In the FEA model, all degrees of freedom were fixed in the clamped edges of the harvesters. With Similar motion degrees of freedom, the MFC transducer was also connected to the beam substrate. C3D20R, a 20-node quadratic brick element, and C3D20RE, a 20-node quadratic piezoelectric brick element, were used to model the beam substrate and the MFC piezoelectric transducer, respectively. Each node possessed three translational degrees of freedom for both elements in the polar coordinate system. After a discretisation and convergence test, the element size was chosen to be kept at 1 mm, sufficient to produce accurate results without requiring excessively long processing time [34]. Table 2, Table 3 present the material properties chosen for the simulation.

#### 2.2.1 Static Analysis

FEA Static Analysis was instrumental in facilitating the authors' comprehension of stress distribution and stress/strain generation within the harvester systems. These parameters assume pivotal significance in designing piezoelectric energy harvesting systems, as the capacity to generate substantial strain inherently translates into higher voltage and enhanced power output. During the analytical phase, a fictitious load of 1 N was applied to the central node at the free end of each device following a similar study conducted by Ref. [34]. For the BSBH, the load was partitioned into two equal portions of 0.5 N, which were subsequently applied to both branches, ensuring equivalence in the 1 N load magnitude with the CBH and SBH configurations.

##### 2.2.1.1 Stress distribution

**Fig. 4:**
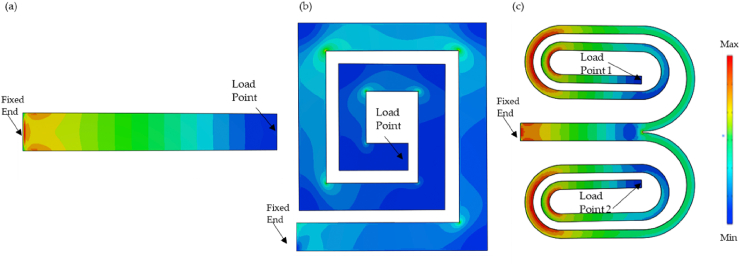
Stress distribution of (a) CBH and (b) SBH (c) BSBH.

Fig. 4 presents the distributed nature of stresses formed in deformed harvesters experiencing 1 N concentrated load. In each device, the warm colours indicate tension, while the cold colours represent compression, with blue indicating zero stress [16]. It is worth noting that the magnitude of stress and strain are different for each design based on their capability of stress/strain generation based on the applied load.

As per Fig. 4 (a), stress generated in the cantilever beam harvester was concentrated near the beam's fixed end. Therefore, when the piezo transducer is attached near the fixed end, it can experience the maximum stress generated in the beam. Fig. 4 (b) presents the stress distribution of SBH. In contrast, the stress distribution of SBH was completely different from that of CBHs as it was unevenly distributed over the structure. This was because, in zig-zag and spiral designs, the low resonance frequency was obtained by reducing the stiffness by distributing stress over the structure [16]. Therefore, a challenge arises when deciding the optimal location for placing the piezoelectric transducer. Berdy, Srisungsitthisunti [35] explained that if the piezoelectric transducer is to be attached along the entire length of the beam, it must be segmented and polarised in opposition to neighbouring sections, owing to the alternating stress polarity.

Moreover, it can be noted in Fig. 4 (b) that as it approaches the beam's free end, the stress magnitude decreases. Consequently, this leads to a scenario where each subsequent piezoelectric segment becomes less effective, and connecting similarly polarised segments in parallel causes adverse charge redistribution, ultimately decreasing overall efficiency [16,36,37]. The second reason for this overall stress distribution is the heavy presence of torsional forces in spiral harvesters [25]. Therefore, the harvester's effectiveness should improve if these forces were fully/partially eliminated or ideally converted them into bending forces. Fig. 4 (c) presents the stress distribution of BSBH. In contrast, the stress distribution of BSBH was more concentrated near the fixed end and has greatly improved the distribution compared to SBH. A possible reason for this significant improvement was introducing two equal branch spiral sections instead of having a single spiral harvester to adopt the symmetry and minimise the effect of torsional forces by allowing more pure bending motion in the structure. To further improve the stress concentration of the BSBH in future studies, as a start-up two identical proof masses can be placed on wing 1 and wing 2 to convert the remaining torsional motions into bending and torsion composite motions. subsequently, the optimal concentrated stress can be studied by varying the position of the masses along the spiral branches.

##### 2.2.1.2 Highest stress and strain

The static analytical investigation elucidates the energy generation potential of each harvester, highlighting that piezoelectric transducers reach their peak voltage output under maximum stress conditions. As depicted in Fig. 4, the highest stress levels are observed near the fixed ends of CBH and BSBH, whereas, for SBH, the peak stress is distributed unevenly at the corners of each turning point throughout the harvester. Table 4 presents the estimated stress and strain on the three beams close to the fixed end, where stress concentration is most significant. The maximum stress near the fixed end of CBH is 4.86E+01 MPa, while the minimum stress is 4.37E+01 MPa. SBH, with its stress dispersed over the harvester, shows minimal stress concentration near the fixed end. However, the maximum stress near the fixed end for SBH is 5.82E+01 MPa, with a minimum of 4.36E+01 MPa. In comparison, BSBH exhibits a significantly higher stress concentration near the fixed end where the MFC transducer is bonded, with maximum and minimum stresses of 1.41E+02 MPa and 1.27E+02 MPa, respectively. For comparative purposes, the average stress values are considered and summarised in Table 4. A similar trend can be observed in the strain distribution of the harvesters near the fixed end. The average strain values are as follows: CBH exhibits a strain of 2.54E-04, SBH shows a strain of 4.14E-04, and BSBH has a strain of 1.83E-03.

**Table 4:** The magnitudes of the stress and strain with the given harvesters.

		Min	Max	Average
CBH	Stress (MPa)	4.37E+01	4.86E+01	4.62E+01
	Strain	2.43E-04	2.65E-04	2.54E-04
SBH	Stress (MPa)	4.36E+01	5.82E+01	5.09E+01
	Strain	3.31E-04	4.96E-04	4.14E-04
BSBH	Stress (MPa)	1.27E+2	1.41E+02	1.34E+02
	Strain	1.73E-03	1.92E-03	1.83E-03

The stress generated by BSBH near the fixed end was approximately 2.5 times greater than that of SBH and 3 times higher than that of CBH. Additionally, the strain produced by BSBH was 4.5 times greater than SBH and 7 times greater than CBH. This significant improvement is likely due to the large deformations caused by the spiral branch beams of BSBH, which amplify the deformation and bending of the main beam. Importantly, the highest stress in BSBH was more concentrated near the fixed end, effectively mitigating the drawbacks observed in SBH. These findings highlight the potential of the proposed BSBH design to generate higher voltage and power under dynamic excitation. It is worth noting that the Ultimate Tensile Strength (UTS) and Tensile Yield Strength (TYS) of the selected aerospace-grade aluminium are 469 MPa and 324 MPa, respectively. Therefore, BSBH can sustain the achieved maximum stress near the fixed end under a hypothetical 1 N loading. However, it is not advisable to attach such a heavy weight to the harvester in practical applications, as it could compromise end-user comfort.

#### 2.2.2 Modal analysis and mode shapes

##### 2.2.2.1 Modal analysis

A linear perturbation analysis was performed in SIMULIA ABAQUS™ to understand each harvester's operating bandwidth and dynamic properties of the first six natural frequencies (NF). The software utilised Equation (1) to solve the eigenvalue problem during the analysis. In this context, ω represented the system's frequency, M represented the mass matrix, ϕ defined the mode shape, and K symbolised the system's stiffness. Table 5 presents the natural frequencies obtained by each harvester.

(1) $$‐\omega^{2}M\phi +K\;\phi =0$$

**Table 5:** First six NFs of each PEH design.

Harvester Type	NF 1 (Hz)	NF 2 (Hz)	NF 3 (Hz)	NF 4 (Hz)	NF 5 (Hz)	NF 6 (Hz)
CBH	22.63	141.71	298.20	397.37	780.83	807.61
SBH	5.00	8.49	10.31	17.64	28.22	39.91
Proposed BSBH	2.94	3.28	4.31	5.56	7.80	7.95

According to the results presented in Table 5, CBH had the narrowest operating bandwidth among all three harvesters, spread over 23 Hz–808 Hz and could not achieve any NF in the desired ultra-low frequency range of 1–10 Hz. In most cases, to reduce the NF of CBH, a massive block mass was used. However, the authors aim to avoid additional penalties in this study, including block masses in the designs. Hence, this narrow operating bandwidth was observed in CBH. Compared to CBH, SBH improved operating bandwidth significantly, widening the working frequency spectrum to 5 Hz–40 Hz. SBH could achieve three NFs in the desired frequency range. Even though the SBH achieved the desired frequency range, it did not satisfy the extremely low-frequency range, such as for aerobic human motions (1 Hz–6 Hz) [19]. SBH had only one NF within the particular region, 4 Hz from the lowest boundary (1 Hz) of aerobic human motion frequency. Since the PEH can produce the maximum output power when the system is excited at its natural frequency, SBH needs further improvements in operating bandwidth to cooperate effectively with lower frequencies source, such as human motion.

On the contrary, the proposed BSBH demonstrated notable improvement compared to CBH and SBH in the ultra-low frequency range, including extremely low human motion frequencies. That's because BSBH attained six NFs within the desired frequency range (<10 Hz), representing a six-times improvement over CBH and a two-times improvement over SBH. When working with human motion vibrations, BSBH improved four times in NFs compared to SBH. It was also noted that in BSBH, the gap between two adjoining NFs had reduced. As stated above, PEHs generate high voltage output at their’ NFs. Hence, reducing the gap between the adjoining NFs will potentially help the harvester generate considerable power output, with frequencies relying on this band gap [19]. This assertion could be further validated during the experimental phase. Overall, the proposed BSBH has a widened operating bandwidth, having six NFs spread over 2.94 Hz–7.95 Hz of the frequency spectrum, addressing one of the primary challenges associated with CBH.

##### 2.2.2.2 Mode shape analysis

**Fig. 5:**
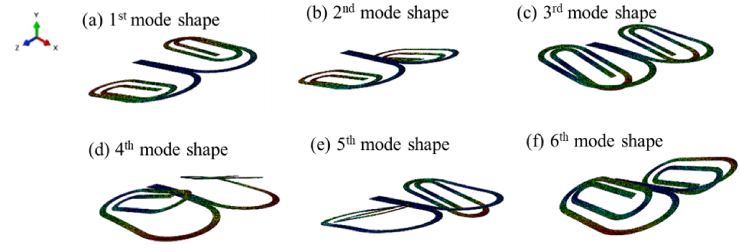
The first six mode shapes obtained from BSBH.

Fig. 5 illustrates the mode shapes of the first six natural frequencies of the proposed BSBH. The motion of BSBH was unidirectional in three-dimensional space. That is similar to the case of a cantilever beam, where the motion was limited to the beam bending along the Y-direction. In human motion, this is favourable to harness energy from the foot strike during the human walking process [38]. In Fig. 5, it can be seen that from the 1st mode shape to the 5th mode shape, the spiral branches undergo higher deflection in their transverse direction (out-of-plane deflection) compared to rotational displacement. Therefore, the behaviour of these modes was considered bending-dominant, with each mode exhibiting its own deformation magnitude. In all these cases, it can be seen that the deformation of two branch spiral beams acts as wings, amplifying the deformation of the main beam. Moreover, the sequential deflection of the spiral branches in the transverse direction across different mode shapes highlights the intricate interplay between the structural geometry and the vibrational modes of the harvester. Compared to the neutral state of BSBH, the highest amplification wings can be seen in the 4th NF among the other NFs. The bending deformation observed in these modes signifies the flexibility and compliance of the spiral branches, which play a crucial role in accommodating mechanical vibrations and facilitating energy conversion. The 6th mode represents a combination of torsion and bending, without any dominant behaviour being evident. As discussed in section 2.2.1.1., SBH is heavily influenced by torsional deformations, leading to distributed stress over the structure [16,26]. By introducing two equal branch spiral sections in BSBH, the new system has adopted the symmetry and minimise the effect of torsional forces by allowing more pure bending motion in the structure, which has led to attaining more concentrated stress near the fixed end of the BSBH as shown in Fig. 4 (c).

### 2.3 Experimental study

A series of experimental tests were carried out to further validate the results obtained by FEA studies. The tests were conducted using a mechanical shaker with controlled impulse acceleration [3] and stable low-frequency vibrations. This consistent impulsive acceleration was produced to simulate typical human movements [3]. Considering most frequent aerobic human exercises/motions of a grown healthy person fall within 1 Hz–6 Hz [39,40], the proposed harvester was tested across 1–6 Hz, with increments of 0.25 Hz. Three readings were taken for each frequency under 0.1 g low acceleration. The experimental outcomes were assessed in terms of operating bandwidth, output voltage, output power, and average power.

According to the design presented in section 2.1, BSBH was fabricated by a computer numerical control (CNC) router machine using an aero-graded aluminium sheet. MFC—M2814P2 transducer was attached near the fixed end of BSBH, which was identified as the highest concentrated stress area using two-part epoxy. No additional penalties, including mass, magnets, and stoppers, have been used in this study. The physical properties of aero-graded aluminium and MFC are detailed in Table 2, Table 3 It's important to highlight that the dimensions of the BSBH harvester were selected as a proof of concept. Given the unique properties of piezoelectric materials, this device can be scaled down to be used as microstructures for real-time applications [41,42].

**Fig. 6:**
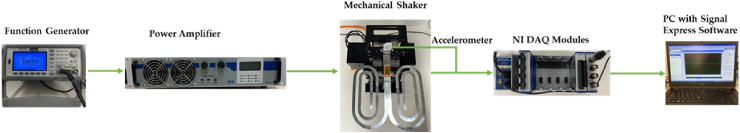
Schematic of the experimental setup.

Fig. 6 displays the diagram illustrating the mechanical shaker test setup. One end of the proposed harvester was affixed to an electrodynamic shaker (APS-113, APS Dynamics, Inc., Dresden, Germany). Under controlled conditions, the BSBH was subjected to excitation using the electrodynamic shaker with a consistent impulse acceleration. The mechanical shaker was operated by supplying the necessary frequency input through the function generator (Agilent 33210A, Santa Clara, CA, USA), with power control managed by a power amplifier (APS125, APS Dynamics, Dresden, Germany). An accelerometer (Dytran 3305A2, 0.3–5000 Hz, ±5 %, Chatsworth, CA, USA) was securely attached to the base of the BSBH to monitor the continuous impulse acceleration. The output voltage was measured using NI DAQ module NI 9229, while the acceleration was measured with NI DAQ module NI 9234. The data were recorded using Signal Express software. The power output (P) was determined by calculating the harvested voltage (V) when the circuit was in an open-circuit condition. As the experiments were performed under open circuit conditions, which approximate infinity resistance, a significantly higher load resistance is needed to compute the power output. Noting that the finite input impedance of the NI DAQ module (NI 9229) was 1 MΩ, to calculate the power out of BSBH, the same 1 MΩ load resistance was considered to satisfy the open circuit condition. Power output and average power output were calculated according to the equations provided in Ref. [19].

## 3 Experimental results and discussion

### 3.1 Output voltage and harvested power

#### 3.1.1 Output voltage

The voltage output of BSBH was measured under 0.1 g acceleration for a frequency sweep of 1–6 Hz, with increments of 0.25 Hz. Based on the evaluations conducted in the FEA study, it was noted that the BSBH demonstrated superior performance compared to the SBH. As a result, the experiments were exclusively directed towards the BSBH, with the SBH being omitted from consideration in the experiments and not presented. However, shaker tests were conducted for the CBH, utilising the same frequency sweep and under similar dynamic conditions, to establish a baseline comparison. The CBH was selected over the SBH because it serves as a representation of the conventional piezoelectric energy harvester commonly employed. Also, SBH did not gain a significant stress/strain near to the fixed end in FEA compared to CBH.

**Fig. 7:**
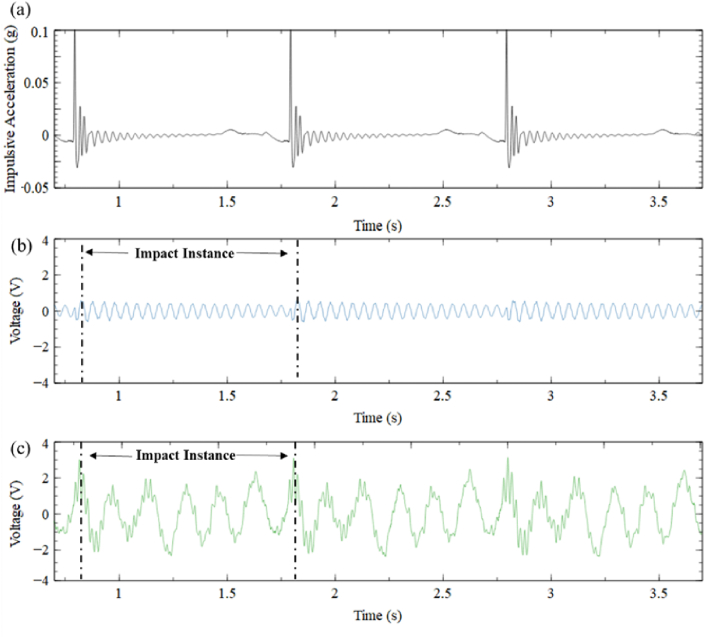
(a) Impulsive base acceleration (b) Voltage output of CBH (c) Voltage output of BSBH.

Fig. 7 present the output voltage attained by CBH (Fig. 7 (b)) and BSBH (7c) when subjected to 0.1 g acceleration within three motion cycles. The highest voltage achieved for CBH and BSBH when subjected to a 0.1 g acceleration was around 0.6 V and 3 V, respectively, which was a five times higher voltage improvement against CBH and agreed with the pattern of the strain analysis results obtained for the two harvesters. Moreover, the first natural frequency of CBH was 22 Hz during the FEA study, which was 21 Hz away from the excited frequency. Therefore, it was expected that CBH would result in lower voltage generation in this instance.

#### 3.1.2 Harvested power

**Fig. 8:**
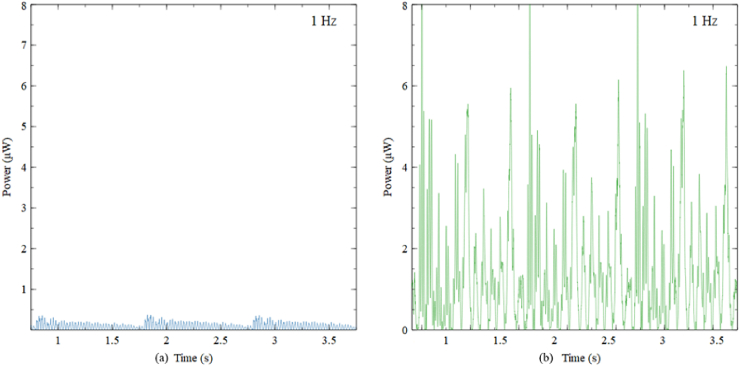
Power generated by (a) CBH and (b) BSBH under 1 Hz and 0.1 g acceleration.

Fig. 8 illustrates the computed output power generated by CBH and BSBH when subjected to a 0.1 g acceleration. Given that the experiments were conducted under open circuit conditions, which can be approximated as having nearly infinite resistance, it was necessary to use a substantially higher load resistance for accurate power calculations using Equation (2) given in Ref. [19]; furthermore, considering that the NI DAQ module (NI 9229) had a finite input impedance of 1 MΩ. Considering these aspects, a load resistance of 1 MΩ was assumed for the power output calculation [3,17,18].

Fig. 8 illustrates that under a 0.1 g acceleration, BSBH generated a maximum power of 8 μW, whereas CBH harvested nearly a maximum power of 0.5 μW. Similar to the voltage generation of the CBH, the power generated by the CBH decreases rapidly during each motion cycle due to increased damping. In contrast, the BSBH consistently generated power ranging from 2.5 to 8 μW throughout the motion cycle. This indicates that the BSBH exhibited greater proficiency in power generation when compared to the CBH. In future research endeavours, it is advisable to employ various resistors to assess the power generation of BSBH. This approach can help to understand the harvester's performance across various loading conditions.

### 3.2 Operating bandwidth

**Fig. 9:**
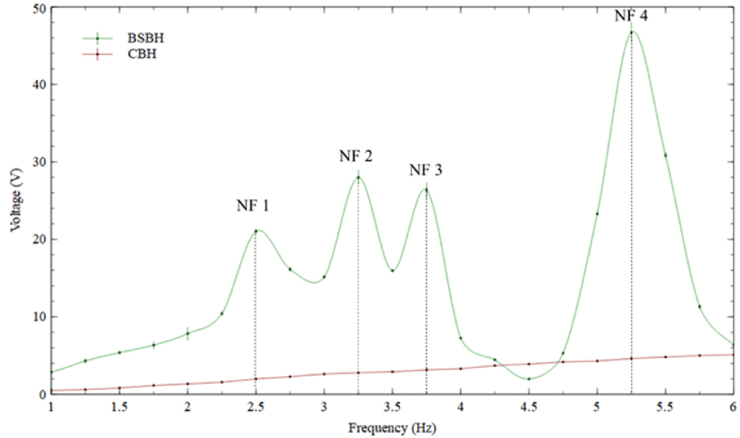
Peak voltage obtained by BSBH and CBH under 0.1 g acceleration.

Assessing a PEH's performance is closely tied to its operational bandwidth, and broadening this bandwidth has the potential to enhance the PEH's efficiency. Consequently, conducting a frequency sweep test ranging from 1 Hz to 6 Hz was crucial to validate the results obtained through FEA analysis. Fig. 9 demonstrates the maximum output voltage achieved for both CBH and BSBH when subjected to a 0.1 g acceleration across 1–6 Hz, with intervals of 0.25 Hz.

According to the data presented in Fig. 9, CBH couldn't achieve any natural frequencies within the frequency range of 1–6 Hz. During the excited frequency sweep, the voltage generated by CBH showed a gradual increase. This was because the first natural frequency of CBH was 22 Hz, and when the excitation frequency gets closer to the natural frequency, the harvester tends to show a gradual increase in voltage [3,43]. However, due to the low stress/strain generation observed in the FEA analysis, the voltage acquired by CBH during the frequency sweep was comparatively low compared to the voltage generation of BSBH.

In contrast, for BSBH, four voltage peaks were observed within the frequency of interest, corresponding to the identified natural frequencies during FEA analysis. Table 6 compares the FEA-derived and experimental natural frequencies for convenient reference. The greatest disparity between the two investigations remained within a margin of 0.56 Hz. Therefore, the results obtained from the experimental tests provide additional evidence of the substantial expansion of the operating bandwidth of the BSBH. Notably, the BSBH exhibits four natural frequencies within the ultra-low frequency spectrum, in contrast to the CBH.

**Table 6:** Table of natural frequencies obtained in FEA study and experimental study.

Type of Analysis	NF 1 (Hz)	NF 2 (Hz)	NF 3 (Hz)	NF 4 (Hz)
FEA Study	2.94	3.28	4.31	5.56
Experimental Study	2.50	3.25	3.75	5.25

Next, with the aid of Fig. 9, the effectiveness of voltage generation at each natural frequency was discussed for BSBH. During the modal analysis in section 2.2.2. it was identified that all the first four natural frequencies of BSBH were bending dominant. Moreover, out of the four modes, it was noted that the 4th bending mode in BSBH experienced the highest displacement. As per the results presented in Fig. 9, the highest voltage output was attained at the fourth natural frequency with a magnitude of 45 V. Therefore, it was preferable to excite the BSBH at its fourth natural frequency to achieve the highest electrical response. The second resonance frequency of BSBH generated the second-highest electrical output of 27 V. When considering the peak voltage of the fourth natural frequency (45 V) as the reference threshold for comparison. Notably, the second natural frequency could only reach 60 % of the threshold voltage during each motion cycle.

Similarly, the third (26 V) and first (21 V) natural frequencies could gain only 57 % and 48 % of the threshold voltage at each motion cycle. These results reflect that the rest of the three can produce approximately 50 % of threshold voltage output when they are dynamically excited. This was a significant outcome for a harvester operating under extremely low (0.1 g) excitation.

#### 3.2.1 Average output power generated by different NFs

**Fig. 10:**
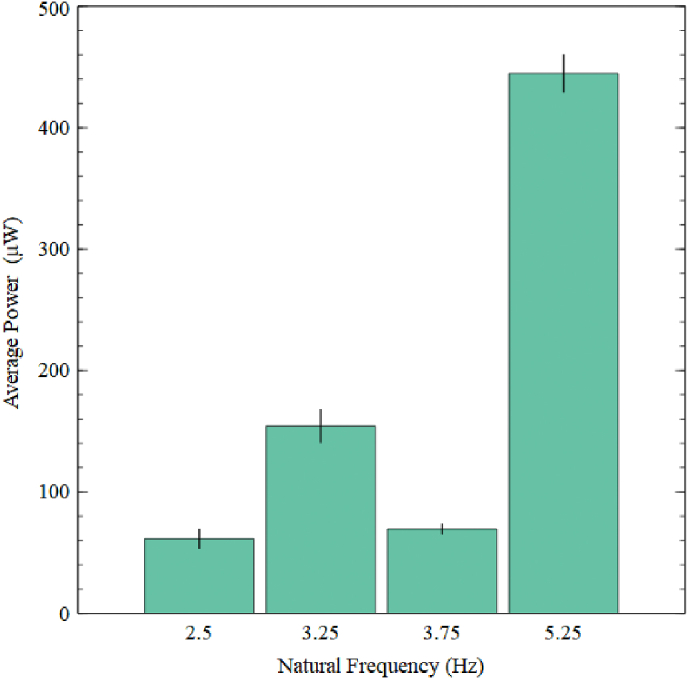
Average output power generated by BSBH at first four natural frequencies.

To further analyse the effectiveness of the proposed design, the average power output was calculated as defined by Equations (3) given in Ref. [19] for each natural frequency obtained by BSBH. The results are presented in Fig. 10. The average power calculations were based on the power generated within 1 s. The least performing natural frequencies were 2.5 Hz and 3.75 Hz, with an average power of 61 μW and 70 μW, respectively. Fig. 9 shows that the voltage at the second natural frequency was slightly incremented (3 V) over the voltage generated at the third natural frequency. However, regarding power generation, the second natural frequency generated 154 μW, nearly two times the average power of both the first and third natural frequencies. This was because the damping of the second natural frequency was lesser than the third natural frequency. Compared to the first three natural frequencies, the fourth natural frequency of BSBH generated a higher average power of 445 μW. This was the best performing natural frequency of BSBH, having 6 times higher average power compared to the first and third natural frequencies while 3 times higher average power than the second natural frequency.

### 3.3 Optimisation of BSBH based on response surface method

In previous sections, the design of the BSBH and its workability were discussed. The proposed harvester, BSBH, was designed to operate efficiently in the ultralow frequency sweep of 1–10 Hz, including vibration sources like human motion. However, most aerobic human exercises/motions of a grown healthy person fall within 1 Hz–6 Hz [19]. Therefore, it is favourable for BSBH to have several natural frequencies within this desired range. With the current geometrical properties, the BSBH can only acquire four natural frequencies within the target frequency range (1 Hz–6 Hz). Hence, to optimise the design parameters to achieve a higher number of natural frequencies (broader operating bandwidth), a statistical technique named Response Surface Method (RSM) was used. RSM is an approach to design experiment models and optimise processes involving multiple variables influencing a specific response or outcome. It comprises a set of statistical and mathematical techniques and offers several advantages compared to conventional and factorial methods [44,45]. Response surface models are built using datasets derived from either physical experiments or computer-based simulations. Two types of design methods, namely, Central Composite Design and Box-Behnken Design, are commonly adopted for the response surface method.

For this study, the Box-Behnken experimental design with five variables, two levels, and a total of 46 experimental runs have been selected. The relationship between the independent and dependent outcome variables is typically approximated using a simplified polynomial model, as shown in Equation (5). $\beta_{i}$ and $x_{i}$ represent the coefficients and variables in the regression model, while ε defines the statistical error term [46,47].

(5) $$y=\beta_{0}+\beta_{1}x_{1}+\beta_{2}x_{2\;}+\ldots +{\;\beta }_{n}x_{n}+\varepsilon$$

when the system exhibits curvature in its response, it is advisable to use a higher-order polynomial model, such as a second-order model (Equation (6)), to capture the non-linear behaviour more accurately. In the regression model, the coefficients $\beta_{0}$, $\beta_{i}$, $\beta_{\text{ii}}$, and $\beta_{\text{ij}}$ correspond to the intercept, linear, quadratic, and interaction terms. The variables $x_{i}$ and $x_{j}$ represent the design parameters in their coded format [46,47].

(6) $$y=\beta_{0}+\sum_{i=1}^{n}\beta_{i}x_{i}+\sum_{i=1}^{n}\beta_{\text{ii}}x_{i}^{2}+\sum_{i<j}^{n}\beta_{\text{ij}}x_{i}x_{j}+\varepsilon$$

**Fig. 11:**
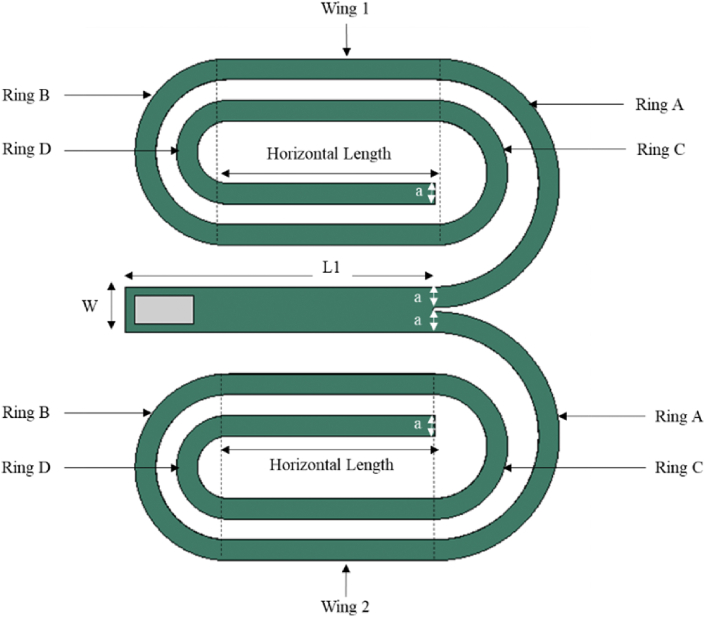
Chosen parameters of BSBH for the optimisation.

Fig. 11 and Table 7 show the input parameters and their corresponding levels used to generate the design of experiment trials for BSBH. As labelled in Fig. 11, the BSBH was divided into two symmetrical sections: Wings 1 and 2. The input parameters considered in the two wings were kept identical to their corresponding to maintain symmetry throughout the study. This way, the analysis can more conveniently illustrate the critical parameters affecting the first and sixth natural frequency of the BSBH. The main beam length (150 mm), thickness (0.06 mm) of the harvester and width (10 mm) of each branch beam were kept as fixed parameters throughout the study. Table 8 summarises the Box-Behnken matrix created using the Minitab program. Each design trial underwent modal analysis in FEA software, and their first and sixth natural frequencies were included in the Box-Behnken matrix as the surface response. The ultimate goal is to optimise BSBH to have this first and sixth natural frequency between 1 Hz–6 Hz.

**Table 7:** Input parameters and their levels of BSBH for the optimisation.

Symbol	Parameter	Levels	
		Low (−1)	High (1)
$x_{1}$	Outer Raduis of Ring A	65	75
$x_{2}$	Outer Raduis of Ring B	45	55
$x_{3}$	Outer Raduis of Ring C	30	40
$x_{4}$	Outer Raduis of Ring D	15	25
$x_{5}$	Horizontal length	50	100

**Table 8:** Experimental design-Box Behnken design matrix.

Specimen no.	Ring A (mm)	Ring B (mm)	Ring C (mm)	Ring D (mm)	Horizontal length (mm)	First NF (Hz)	Sixth NF (Hz)
1	65	45	35	20	75	3.2825	8.5908
2	75	45	35	20	75	2.7661	8.1916
3	65	55	35	20	75	3.1968	7.1247
4	75	55	35	20	75	2.7348	6.6360
5	70	50	30	15	75	3.0981	7.6432
6	70	50	40	15	75	2.9686	7.5684
7	70	50	30	25	75	3.0786	7.4277
8	70	50	40	25	75	2.8854	7.4176
9	70	45	35	20	50	3.5765	10.5650
10	70	55	35	20	50	3.5576	8.1341
11	70	45	35	20	100	2.4038	6.8548
12	70	55	35	20	100	2.2342	5.5566
13	65	50	30	20	75	3.3133	7.7576
14	75	50	30	20	75	2.8140	7.3452
15	65	50	40	20	75	3.1834	7.7471
16	75	50	40	20	75	2.6982	7.3075
17	70	50	35	15	50	3.6334	9.3267
18	70	50	35	25	50	3.5308	9.0453
19	70	50	35	15	100	2.4628	6.4562
20	70	50	35	25	100	2.3866	6.1672
21	70	45	30	20	75	3.0812	8.4159
22	70	55	30	20	75	2.9968	6.9143
23	70	45	40	20	75	2.9607	8.3227
24	70	55	40	20	75	2.9049	6.8465
25	65	50	35	15	75	3.3073	7.8559
26	75	50	35	15	75	2.7977	7.4323
27	65	50	35	25	75	3.2005	7.6575
28	75	50	35	25	75	2.7213	7.2290
29	70	50	30	20	50	3.6662	9.2266
30	70	50	40	20	50	3.4860	9.1267
31	70	50	30	20	100	2.4626	6.3198
32	70	50	40	20	100	2.3808	6.2828
33	65	50	35	20	50	3.8454	9.4213
34	75	50	35	20	50	3.2651	9.0388
35	65	50	35	20	100	2.6082	6.6578
36	75	50	35	20	100	1.9868	5.5211
37	70	45	35	15	75	3.0506	8.4621
38	70	55	35	15	75	3.0021	6.9210
39	70	45	35	25	75	2.9660	8.2425
40	70	55	35	25	75	2.9073	6.7482
41	70	50	35	20	75	2.9946	7.5382
42	70	50	35	20	75	2.9946	7.5382
43	70	50	35	20	75	2.9946	7.5382
44	70	50	35	20	75	2.9946	7.5382
45	70	50	35	20	75	2.9946	7.5382
46	70	50	35	20	75	2.9946	7.5382

#### 3.3.1 Response analysis

**Fig. 12:**
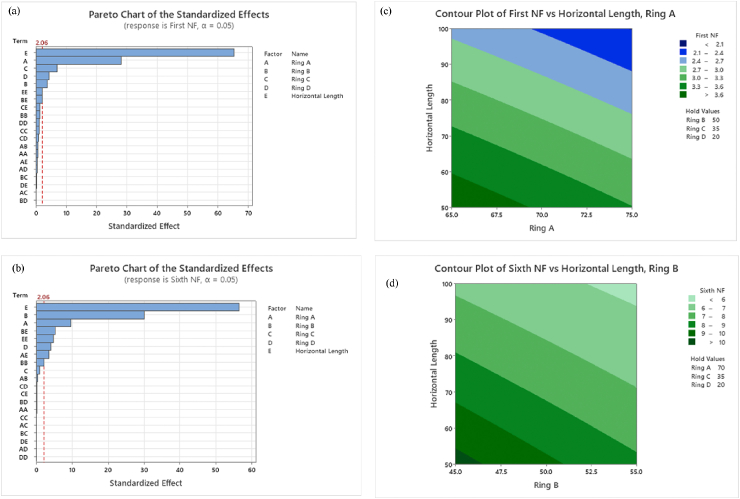
Critical parameters affecting on natural frequencies of BSBH (a) Pareto Chart for 1st NF, (b) Pareto Chart for 6th NF, (c) Contour plot of 1st NF and (d) Contour plot of 6th NF.

The relationships between the natural frequencies and the input parameters affecting them are presented in the format of Pareto charts and shown in Fig. 12 (a) and 12 (b). As per the results, the input parameters exceed the 2.06 standardised effect, critically affecting the magnitude of given natural frequencies. The horizontal length and radius of Ring A have a significant effect on the first natural frequency, while the horizontal length and radius of Ring B significantly affect the sixth natural frequency. This can be further explained using the contour plots illustrated in Fig. 12 (c) and 12 (d). Each contour plot demonstrates the impacts of the two critical variables within their respective investigated ranges while keeping the remaining three variables fixed at their average value (zero level) of the given low and high levels in Table 7. These plots help assess the influence of the main factors and their interactions on the desired response. The results demonstrate that the lowest first natural frequency was obtained when the horizontal length of the branches and the radius of Ring A were at their highest level. Concurrently, the lowest sixth natural frequency (<6 Hz) was achieved when the length of the horizontal branches and the radius of Ring B were at their maximum level. Moreover, it is evident that simultaneously altering these three variables led to a reduction in both natural frequencies, indicating a mutual interaction effect between the horizontal length of the radius of the branches of Ring A and Ring B.

#### 3.3.2 Optimisation

During this phase, the first and sixth natural frequencies were optimised using the “Response Optimiser” feature within Minitab software. This feature optimises the Box Behnken designs given in Table 8 to achieve a given target. As stated above, this study aims to achieve as many natural frequencies as possible within the 1–6 Hz range for BSBH (due to most aerobic human exercises/motions of a grown healthy person fall within the particular range). Therefore, the selected target frequency range for the optimisation was 2–5 Hz, which fits into the desired frequency range. This selection was made since all the considered aerobic movements could achieve at least one of the frequencies in the target frequency range, as outlined in Table 9.

**Table 9:** Frequency ranges for typical aerobic human motions.

Reference	Human Motion Type	Frequency Range of Interest (Hz)
Cai, Yang [39]	Walking	0.5–2
Pańtak [48] & Tan, Zhang [49]	Jogging	1–3
Cavagna, Willems [40]	Running	1–5

Minitab software recommended an optimised set of geometrical properties for the BSBH, as detailed in Table 10, to attain the targeted frequencies (2 Hz and 5 Hz).

**Table 10:** Optimised parameters for the target frequencies (i.e. 2 Hz and 5 Hz).

Solution	Ring A (mm)	Ring B (mm)	Ring C (mm)	Ring D (mm)	Horizontal Length (mm)	First NF nearest Fit (Hz)	Sixth NF nearest Fit (Hz)
1	75	55	34.7968	25	100	2	5

The recommended optimised specimen was tested using FEA software and in a physical experiment. It is worth noting that the radius of Ring C suggested by the optimisation method was rounded off to the nearest 35 mm for FEA and experimental testing purposes. The results obtained by both studies (FEA and experimental tests) have been concluded in Table 11.

**Table 11:** Natural frequencies of the optimised BSBH.

Type of Analysis	NF 1 (Hz)	NF 2 (Hz)	NF 3 (Hz)	NF 4 (Hz)	NF 5 (Hz)	NF 6 (Hz)
FEA Study	2.07	2.18	3.24	4.09	5.15	5.20
Experimental Study	1.25	2.00	2.75	4.00	4.75	5.75

With the results summarised in Table 11 and it can be concluded that the FEA and experimental results satisfactorily match the given targets to the “Response Optimiser”. The greatest disparity between the two investigations remained within a margin of 0.82 Hz. Hence, the dimensions suggested by the response optimiser are suitable for achieving common aerobic human motion frequencies outlined in Table 9. These dimensions can be scaled to suit the application using PEH scaling laws outlined in Refs. [41,42]. Furthermore, the suggested optimised BPEH parameters improve the operating bandwidth of the harvester, having six natural frequencies in a target frequency range (1–6 Hz), where it previously had only four natural frequencies (Refer to Table 5).

In summary, the results obtained throughout the study prove that BSBH successfully diminishes the current drawbacks in spiral harvesters (i.e. being a concentrated stress structure with wider operating bandwidth) and has the ability to power low-power consuming WSNs (<100 μW) with a suitable rectifier circuit. For practical applications, this optimised BSBH, designed as an uni-directional harvester for human motion, is recommended for testing with vibrations from heel strikes integrated into flooring. To implement it, a durable floor tile system with rectifier and boost converter circuits needs to be designed, suitable for high foot traffic areas. The generated system can subsequently power nearby smart systems, including automated pedestrian gates.

## 4 Conclusions

This study aimed to enhance the performance of multimodal PEH designs driven by ultra-low frequency vibrations, incorporating the advantages of spiral and branch beam concepts together. A numerical investigation was conducted using SIMULIA ABAQUSTM FEA software, complemented by a series of experimental tests, to evaluate the performance of the proposed BSBH across parameters, including operating bandwidth, voltage generation, and power generation. The motivation behind the study was from literature as it emphasised that conventional spiral structures exhibit torsional forces that diminish their stress-generation ability. This study proposed a characteristic spiral harvester design to eliminate these torsional forces by introducing symmetry to the system. The aforementioned symmetry was introduced by incorporating the branch beam concept into the overall design. The objective was achieved by having a concentrated stress structure, where stress in the beam is focused in a confined area suitable for placing a piezoelectric layer, as opposed to being distributed across the entire beam. Introducing the branch beam concept was beneficial for bringing symmetry to the system and helped widen the operating bandwidth while maintaining a set of low resonance frequencies (1–10 Hz). To further improve the stress concentration and operating bandwidth of the proposed design, two identical proof masses can be placed on wing 1 and wing 2 to convert the remaining torsional motions into bending and torsion composite motions. subsequently, the optimal concentrated stress and the bandwidth can be studied by varying the position of the masses along the spiral branches. The investigation also revealed that the maximum output may not necessarily occur at the fundamental resonance frequency but might manifest at higher-order resonances. To further improve the stress concentration and operating bandwidth of the proposed design, two identical proof masses can be placed on wing 1 and wing 2 to convert the remaining torsional motions into bending and torsion composite motions. subsequently, the optimal concentrated stress and the bandwidth can be studied by varying the position of the masses along the spiral branches.

Once the workability of the device was proved, a statistical optimisation method, the “Box-Behnken model,” was introduced to optimise the proposed design. Notably, most of the previously proposed statistical methods for PEH systems were more concentrated on proposing DOE. The current study proposes the “Box-Behnken model” to optimise the design to work in targeted frequencies of 2–5 Hz in an ultra-low frequency range. The results obtained by the statistical optimisation method satisfactorily match the FEA and experimental results. The optimised BSBH could achieve six natural frequencies within 2–5 Hz, favouring common aerobic human motions, including walking, jogging and running. Given the unique properties of piezoelectric materials, this device can be scaled down to be used as microstructures for real-time applications. When coupled with an appropriate rectifier circuit for efficient power management, the BSBH can be an autonomous energy source for low-power devices like medical implants and Wireless Sensor Networks (WSNs <100 μW). In future endeavours, focus can be placed on active or passive tunable methods, having flexi joints for the wings of the harvester to resemble the motion of a butterfly flapping fully. Furthermore, optimisations based on maximum power output can be explored for both active and passive BSBH configurations. Moreover, as an uni-directional harvester optimised for human motion applications, a scaled BSBH can be tested with vibrations from heel strikes, whether integrated into the sole of a shoe or fixed in flooring.

## Funding

This research was supported by10.13039/501100001770Southern Cross University, Australia.

## Data availability statement

Data could be provided upon request to the corresponding author.

## CRediT authorship contribution statement

**Iresha Erangani Piyarathna:** Writing – original draft, Investigation, Formal analysis, Data curation, Conceptualization. **Mustafa Ucgul:** Writing – review & editing, Supervision, Methodology. **Charles Lemckert:** Writing – review & editing, Supervision, Methodology. **Zi Sheng Tang:** Writing - review & editing, Supervision, Methodology. **Yee Yan Lim:** Writing – review & editing.

## Declaration of competing interest

The authors declare that they have no known competing financial interests or personal relationships that could have appeared to influence the work reported in this paper.
